# Association between continuity of care and subsequent hospitalization and mortality in patients with mood disorders: Results from the Korea National Health Insurance cohort

**DOI:** 10.1371/journal.pone.0207740

**Published:** 2018-11-19

**Authors:** Woorim Kim, Suk-Yong Jang, Tae-Hoon Lee, Joo Eun Lee, Eun-Cheol Park

**Affiliations:** 1 Department of Public Health, Graduate School, Yonsei University, Seoul, Republic of Korea; 2 Institute of Health Services Research, Yonsei University, Seoul, Republic of Korea; 3 Department of Preventive Medicine, Eulji University College of Medicine, Daejeon, Republic of Korea; 4 Health Insurance Review and Assessment Service, Wonju, Republic of Korea; 5 Ajou University School of Medicine, Suwon, Gyeonggi-do, Republic of Korea; 6 Department of Preventive Medicine, Yonsei University College of Medicine, Seoul, Republic of Korea; University of British Columbia, CANADA

## Abstract

Concerns have been raised about the loss of treatment continuity in unipolar and bipolar depressive disorder patients as continuity of care (COC) may be associated with patient outcomes. This study aimed to examine the relationship between COC and subsequent hospitalization, all-cause mortality, and suicide mortality in individuals with unipolar and bipolar disorder. Data were from the National Health Insurance (NHI) cohort, 2002 to 2013. Study participants included individuals first diagnosed with unipolar depressive disorder or bipolar affective disorder. The independent variable was COC for the first year of outpatient visits after diagnosis, measured using the usual provider of care (UPC) index. The dependent variables were hospitalization in the year after COC measurement, all-cause mortality, and suicide mortality. Analysis was conducted using logistic regression and Cox proportional hazards survival regression. A total of 48,558 individuals were analyzed for hospitalization and 48,947 for all-cause and suicide mortality. Compared to the low COC group, the medium [odds ratio (OR) 0.30, 95 percent confidence interval (95% CI) 0.19–0.47] and the high COC group (OR 0.14, 95% CI 0.09–0.21) showed statistically significant decreased odds of hospitalization. Additionally, lower likelihoods of suicide death were found in the high (HR 0.35, 95% CI 0.16–0.74) compared to the low COC group. The results infer an association between COC after first diagnosis of unipolar or bipolar depressive disorder and hospitalization and suicide mortality, suggesting the potential importance of treatment continuity in improving patient outcomes.

## Introduction

Depression is a leading cause of disability worldwide that has cost over United States (US) four billion societal costs in South Korea [1, 2]. Specifically, major depressive disorder is a multifaceted mental disorder that includes a wide range of symptoms related to the functioning of the mood, cognition, and motor, with psychotic experiences and bipolar spectrum features being commonly found in affected patients [3]. In Korean adults, the prevalence of depression was 6.7 percent and that of major depressive disorder 2.7 percent in 2014 [4]. Considering that individuals with major depressive disorder often report higher rates of comorbidity and mortality, reduced quality of life, lower productivity, and higher utilization of health care services, it is important to identify the factors associated with symptom severity in countries where the prevalence of mood disorder is escalating [5].

Due to the increasing prevalence of mood disorder, concerns have been specifically raised about loss of continuity and fragmentation in the care received by mental disorder patients [6]. Continuity of care (COC) is the process by which the patient and the provider maintain a sustained partnership toward effectively meeting the patient’s healthcare needs [7]. COC is particularly central in mental health care as it is linked with improved quality of life and community functioning, reduced symptom severity, higher health service satisfaction, and lower health care costs [8, 9]. Better continuity of care has also been reported to be associated with improved physician-patient relationship, higher patient compliance and satisfaction, and fewer hospital admissions [10, 11].

Previous studies focusing on Western countries have documented that better continuity of care may be important in improving the outcomes of major depressive disorder patients [7, 11]. In fact, studies have specifically reported that maintaining good continuity of care can be related to reduced mortality risk in patients with bipolar or major depressive disorders [8, 12]. Additionally, although studies investigating the relationship between continuity of care and patient outcomes in East Asian countries are comparatively scarce, the related findings did suggest that depressive symptoms may be a predictor of subsequent hospital admissions in Chinese and Singaporean individuals [13]. However, implications from these studies have been limited as only individuals aged 55 or above were incorporated with a follow up time of 12 months or below [13].

Under such circumstances, the aim of this study was to examine the relationship between COC and subsequent hospitalization and all-cause and suicide mortality in individuals diagnosed with unipolar disorder and bipolar depressive disorder using nationally representative data from the Korean National Health Insurance (NHI) cohort. The hypothesis was that better COC will be associated with lower likelihoods of hospital admission, all-cause mortality, and suicide mortality. In addition, subgroup analysis was conducted by type of disease (unipolar or bipolar disorder) and medical institution visited (tertiary hospital, secondary hospital, or primary clinic) in the analysis on COC and hospitalization, particularly because Korea lacks a stable general practitioner system and patients are able to freely choose medical institutions without referrals.

## Materials and methods

### Data source and study population

Data were from the 2002 to 2013 Korea National Health Insurance (NHI) cohort. In Korea, all individuals are covered by the NHI or Medical Aid and the NHI is known to cover around 98 percent of the total population. The NHI cohort is composed of 1,025,340 nationally representative random samples of the Korean population in 2002, which equals around 2.2 percent of the entire population. Data were collected using a systematic sampling method to construct a representative sample of the 46,605,433 residents recorded by the Korean National Health Insurance Service (KNHIS). Follow up data were available up to 2013 and include information on medical claims filed between 2002 and 2013. All personal information in this data were de-identified by the KNHIS before distribution. Data can be utilized after application and approval on the KNHIS website.

Of the 1,025,340 individuals recorded at the baseline, all individuals primarily diagnosed of unipolar depressive disorder (International Classification of Diseases version 10 [ICD-10] F30, F32, F34, F38, and F39) and bipolar affective disorder (F31) were included. Study participants measured in 2002 were excluded to ensure the inclusion of only individuals first diagnosed with unipolar or bipolar disorder. Hence, individuals were followed from 2003 to 2013. All individuals aged 19 or below were also excluded to limit the study population to adults as children and adolescents may exhibit different patterns. Additionally, individuals with less than 3 yearly outpatient visits to physicians were omitted to ensure a stable measurement of COC. This led to the final inclusion of 48,558 cases at risk of hospitalization after excluding those who died during and within one year of COC measurement and 48,947 cases at risk of all-cause and suicide mortality.

### Outcome measures

The dependent variables of this study were hospital admission, all-cause mortality, and suicide mortality. As individuals diagnosed in 2002 were excluded to ensure the inclusion of only patients first diagnosed with unipolar or bipolar disorder, participants were followed from 2003 to 2013 for measurement. Hospital admission was limited to cases with a primary diagnosis of mental disorder recorded within one year after COC measurement. All emergency department visits were excluded from the analysis in which hospitalization was the primary end point. All-cause and suicide deaths were recorded in the NHI cohort based on the database of the National Statistical Office (NSO), which compulsorily receives all reports on death through an official death notice. Suicide mortality was separately identified based on the ICD-10 code X60-84.

### Independent variable

The independent variable of this study was COC measured within one year of initial diagnosis. COC was measured using the usual provider of care (UPC) index. The UPC index is based on density type and is defined as the number of outpatient visits to the most frequently seen physician divided by the total number of outpatient visits [14]. Accordingly, the UPC index focusses on the number of physicians seen by a patient and the visit ratio of the most frequently seen physician to all visited physicians. Values range between zero and one. COC was categorized into the low (≤0.4), medium (>0.4, <0.75), and high (≥0.75) groups based on previous references [15, 16].

### Covariates

Demographic, socioeconomic, and health related covariates were incorporated in this study. Included covariates were frequency of outpatient visits (low or high), diagnosis (unipolar or bipolar disorder), age at diagnosis (20–39, 40–59, 60–79, or 80 or above), sex (men or women), income (low, middle, or high), region (Seoul, urban, or rural), antidepressant (no or yes), antipsychotic (no or yes), anxiolytic (no or yes), stabilizer (no or yes), psychotherapy (none, personal therapy, group therapy, or others), comorbidities measured using the Charlson Comorbidity Index (zero, one, two, three, or four and above), and type of medical institution visited for outpatient services (tertiary hospital, secondary hospital, or primary clinic).

### Analytic approach

The general characteristics of the study participants were examined using chi-square test to compares differences between groups. Hospital admissions in the subsequent year of COC measurement were analyzed using logistic regression analysis, expressed as odds ratio (OR) and their 95 percent confidence intervals (95% CI). Subgroup analysis was performed by type of medical institution visited for outpatient services and type of disease. The association between COC and the likelihood of all-cause and suicide mortality was tested using Cox proportional hazards survival regression analysis, expressed as hazard ratio (HR) and their 95% CI. Analysis was adjusted for all covariates and the calculated P values were two sided, considered significant at <0.05. Analysis was performed using the SAS software, version 9.4 (SAS Institute, Cary, NC, USA).

## Results

The general characteristics of the study participants are shown in Table 1. Of the 48,558 individuals at risk of hospitalization, 152 individuals were categorized into the low, 4,273 into the medium, and 44,133 into the high COC group. A total of 1,201 (2.5%) participants experienced hospitalization. Regarding all-cause and suicide mortality, 48,947 individuals at risk were analyzed. In this sample set, the low COC group included 152 individuals, the medium COC group 4,308 individuals, and the high COC group 44,487 individuals. The overall all-cause mortality rate was 6.4% and the suicide mortality rate 1.1%.

**Table 1:** Characteristics of study participants.

	N	Admission				P-value	N	All-cause mortality				P-value	Suicide mortality				P-value
		No		Yes				No		Yes			No		Yes		
**COC measure**																	
** **Low	152	125	(82.2)	27	(17.8)	< .0001	152	138	(90.8)	14	(9.2)	0.017	145	(95.4)	7	(4.6)	< .0001
** **Medium	4273	3994	(93.5)	279	(6.5)		4308	3994	(92.7)	314	(7.3)		4202	(97.5)	106	(2.5)	
** **High	44133	43238	(98.0)	895	(2.0)		44487	41676	(93.7)	2811	(6.3)		44082	(99.1)	405	(0.9)	
**Outpatient visits**																	
** **Low	9602	9495	(98.9)	107	(1.1)	< .0001	9680	9159	(94.6)	521	(5.4)	< .0001	9620	(99.4)	60	(0.6)	< .0001
** **High	38956	37862	(97.2)	1094	(2.8)		39267	36649	(93.3)	2618	(6.7)		38809	(98.8)	458	(1.2)	
**Diagnosis**																	
** **Unipolar disorder	46708	45694	(97.8)	1014	(2.2)	< .0001	47073	44088	(93.7)	2985	(6.3)	0.0011	46592	(99.0)	481	(1.0)	< .0001
** **Bipolar disorder	1850	1663	(89.9)	187	(10.1)		1874	1720	(91.8)	154	(8.2)		1837	(98.0)	37	(2.0)	
**Age**																	
** **20–39	15522	15082	(97.2)	440	(2.8)	0.0058	15550	15333	(98.6)	217	(1.4)	< .0001	15419	(99.2)	131	(0.8)	0.0006
** **40–59	18830	18394	(97.7)	436	(2.3)		18913	18341	(97.0)	572	(3.0)		18718	(99.0)	195	(1.0)	
** **60–79	12849	12553	(97.7)	296	(2.3)		13041	11262	(86.4)	1779	(13.6)		12866	(98.7)	175	(1.3)	
** **80 or above	1357	1328	(97.9)	29	(2.1)		1443	872	(60.4)	571	(39.6)		1426	(98.8)	17	(1.2)	
**Sex**																	
** **Men	15224	14838	(97.5)	386	(2.5)	0.5513	15430	13895	(90.1)	1535	(10.0)	< .0001	15165	(98.3)	265	(1.7)	< .0001
** **Women	33334	32519	(97.6)	815	(2.4)		33517.0	31913	(95.2)	1604	(4.8)		33264	(99.3)	253	(0.8)	
**Income**																	
** **Low	14067	13772	(97.9)	295	(2.1)	0.0029	14182	13272	(93.6)	910	(6.4)	< .0001	14044	(99.0)	138	(1.0)	0.4335
** **Middle	19069	18571	(97.4)	498	(2.6)		19205	18125	(94.4)	1080	(5.6)		18990	(98.9)	215	(1.1)	
** **High	15422	15014	(97.4)	408	(2.7)		15560	14411	(92.6)	1149	(7.4)		15395	(98.9)	165	(1.1)	
**Region**																	
** **Seoul	10337	10086	(97.6)	251	(2.4)	0.5887	10420	9805	(94.1)	615	(5.9)	0.0296	10310	(98.9)	110	(1.1)	0.8587
** **Urban	11815	11535	(97.6)	280	(2.4)		11909	11154	(93.7)	755	(6.3)		11788	(99.0)	121	(1.0)	
** **Rural	26406	25736	(97.5)	670	(2.5)		26618	24849	(93.4)	1769	(6.7)		26331	(98.9)	287	(1.1)	
**Antidepressant**																	
** **No	44936	43799	(97.5)	1137	(2.5)	0.0044	45306	42363	(93.5)	2943	(6.5)	0.0084	44813	(98.9)	493	(1.1)	0.0227
** **Yes	3622	3558	(98.2)	64	(1.8)		3641	3445	(94.6)	196	(5.4)		3616	(99.3)	25	(0.7)	
**Antipsychotic**																	
** **No	48296	47105	(97.5)	1191	(2.5)	0.1603	48680	45554	(93.6)	3126	(6.4)	0.3017	48163	(98.9)	517	(1.1)	0.2736
** **Yes	262	252	(96.2)	10	(3.8)		267	254	(95.1)	13	(4.9)		266	(99.6)	1	(0.4)	
**Anxiolytic**																	
** **No	45728	44598	(97.5)	1130	(2.5)	0.9003	46089	43148	(93.6)	2941	(6.4)	0.2469	45604	(99.0)	485	(1.1)	0.6039
** **Yes	2830	2759	(97.5)	71	(2.5)		2858	2660	(93.1)	198	(6.9)		2825	(98.9)	33	(1.2)	
**Stabilizer**																	
** **No	48302	47116	(97.5)	1186	(2.5)	0.0005	48690	45574	(93.6)	3116	(6.4)	0.0961	48178	(99.0)	512	(1.1)	0.0450
** **Yes	256	241	(94.1)	15	(5.9)		257	234	(91.1)	23	(9.0)		251	(97.7)	6	(2.3)	
**Psychotherapy**																	
** **None	29408	28710	(97.6)	698	(2.4)	0.0212	29653	27842	(93.9)	1811	(6.1)	< .0001	29381	(99.1)	272	(0.9)	< .0001
** **Personal therapy	18530	18053	(97.4)	477	(2.6)		18668	17410	(93.3)	1258	(6.7)		18432	(98.7)	236	(1.3)	
** **Group therapy	512	491	(95.9)	21	(4.1)		518	460	(88.8)	58	(11.2)		513	(99.0)	5	(1.0)	
** **Others	108	103	(95.4)	5	(4.6)		108	96	(88.9)	12	(11.1)		103	(95.4)	5	(4.6)	
**Charlson Comorbidity Index**																	
** **0	33010	32177	(97.5)	833	(2.5)	0.3346	33223	31622	(95.2)	1601	(4.8)	< .0001	32895	(99.0)	328	(1.0)	0.0155
** **1	5419	5288	(97.6)	131	(2.4)		5478	4968	(90.7)	510	(9.3)		5420	(98.9)	58	(1.1)	
** **2	6559	6419	(97.9)	140	(2.1)		6621	6120	(92.4)	501	(7.6)		6541	(98.8)	80	(1.2)	
** **3	2553	2482	(97.2)	71	(2.8)		2594	2251	(86.8)	343	(13.2)		2551	(98.3)	43	(1.7)	
** **4+	1017	991	(97.4)	26	(2.6)		1031	847	(82.2)	184	(17.9)		1022	(99.1)	9	(0.9)	
**Type of medical institution**																	
** **Tertiary hospital	12797	12163	(95.1)	634	(5.0)	< .0001	12941	11859	(91.6)	1082	(8.4)	< .0001	12721	(98.3)	220	(1.7)	< .0001
** **Secondary hospital	3514	3357	(95.5)	157	(4.5)		3567	3250	(91.1)	317	(8.9)		3523	(98.8)	44	(1.2)	
** **Primary clinic	32247	31837	(98.7)	410	(1.3)		32439	30699	(94.6)	1740	(5.4)		32185	(99.2)	254	(0.8)	
**Total**	48558	47357	(97.5)	1201	(2.5)		48947	45808	(93.6)	3139	(6.4)		48429	(98.9)	518	(1.1)	

Table 2 presents the results of the logistic regression analysis investigating the association between COC and hospitalization in the subsequent year of COC measurement. Compared to the low COC group, the medium (OR 0.30, 95% CI 0.19–0.47) and the high COC group (OR 0.14, 95% CI 0.09–0.21) showed statistically significant lower odds of hospitalizations. The results of the Cox proportional hazards survival regression analysis studying the relationship between COC and all-cause and suicide mortality are also presented on Table 2. The association between COC and all-cause mortality did not show statistical significance. However, individuals with high COC (HR 0.35, 95% CI 0.16–0.74) showed statistically significant decreased likelihoods of suicide death than individuals with low COC.

**Table 2:** Factors associated with hospital admissions, all-cause mortality, and suicide mortality.

	Admission*				All-cause*				Suicide*			
	OR	95% CI			HR	95% CI			HR	95% CI		
**COC measure**												
** **Low	1.00				1.00				1.00			
** **Medium	0.30	(0.19	-	0.47)	1.16	(0.68	-	1.98)	0.74	(0.34	-	1.61)
** **High	0.14	(0.09	-	0.21)	1.14	(0.67	-	1.94)	0.35	(0.16	-	0.74)
**Outpatient visits**												
** **Low	1.00				1.00				1.00			
** **High	2.04	(1.66	-	2.50)	1.06	(0.96	-	1.16)	1.51	(1.14	-	1.99)
**Diagnosis**												
** **Unipolar disorder	1.00				1.00				1.00			
** **Bipolar disorder	3.56	(2.99	-	4.23)	1.31	(1.11	-	1.54)	1.40	(0.99	-	1.99)
**Age**												
** **20–39	1.00				1.00				1.00			
** **40–59	0.80	(0.70	-	0.92)	2.10	(1.80	-	2.46)	1.18	(0.94	-	1.48)
** **60–79	0.74	(0.63	-	0.87)	10.49	(9.08	-	12.12)	1.69	(1.33	-	2.15)
** **80 or above	0.72	(0.49	-	1.07)	48.45	(41.24	-	56.93)	2.08	(1.23	-	3.53)
**Sex**												
** **Men	1.00				1.00				1.00			
** **Women	1.09	(0.96	-	1.23)	0.45	(0.42	-	0.48)	0.42	(0.35	-	0.50)
**Income**												
** **Low	1.00				1.00				1.00			
** **Middle	1.21	(1.04	-	1.40)	0.86	(0.78	-	0.94)	1.09	(0.88	-	1.35)
** **High	1.23	(1.05	-	1.44)	0.83	(0.76	-	0.91)	0.95	(0.75	-	1.19)
**Region**												
** **Seoul	1.00				1.00				1.00			
** **Urban	1.03	(0.87	-	1.23)	1.13	(1.01	-	1.25)	1.02	(0.79	-	1.34)
** **Rural	1.09	(0.93	-	1.26)	1.06	(0.96	-	1.16)	1.08	(0.86	-	1.35)
**Antidepressant**												
** **No	1.00				1.00				1.00			
** **Yes	0.69	(0.53	-	0.89)	0.82	(0.71	-	0.96)	0.70	(0.47	-	1.06)
**Antipsychotic**												
** **No	1.00				1.00				1.00			
** **Yes	0.64	(0.33	-	1.23)	0.59	(0.34	-	1.02)	0.24	(0.03	-	1.71)
**Anxiolytic**												
** **No	1.00				1.00				1.00			
** **Yes	0.86	(0.67	-	1.10)	0.94	(0.81	-	1.08)	0.98	(0.68	-	1.41)
**Stabilizer**												
** **No	1.00				1.00				1.00			
** **Yes	0.92	(0.53	-	1.61)	1.54	(1.02	-	2.32)	1.53	(0.67	-	3.47)
**Psychotherapy**												
** **None	1.00				1.00				1.00			
** **Personal therapy	0.99	(0.88	-	1.12)	0.98	(0.91	-	1.06)	1.17	(0.98	-	1.39)
** **Group therapy	2.03	(1.29	-	3.20)	1.45	(1.11	-	1.88)	1.06	(0.44	-	2.58)
** **Others*†*	1.46	(0.58	-	3.69)	1.21	(0.68	-	2.14)	4.15	(1.70	-	10.13)
**Charlson Comorbidity Index**												
** **0	1.00				1.00				1.00			
** **1	0.91	(0.75	-	1.11)	1.16	(1.05	-	1.28)	0.92	(0.69	-	1.22)
** **2	0.85	(0.70	-	1.02)	0.97	(0.88	-	1.08)	1.03	(0.80	-	1.33)
** **3	1.11	(0.86	-	1.44)	1.29	(1.14	-	1.45)	1.25	(0.89	-	1.75)
** **4+	1.10	(0.73	-	1.65)	1.50	(1.29	-	1.76)	0.67	(0.34	-	1.30)
**Type of medical institution**												
** **Tertiary hospital	1.00				1.00				1.00			
** **Secondary hospital	0.94	(0.78	-	1.13)	1.08	(0.95	-	1.23)	0.78	(0.56	-	1.10)
** **Primary clinic	0.28	(0.25	-	0.32)	0.73	(0.68	-	0.79)	0.53	(0.44	-	0.64)
**Year**	0.96	(0.95	-	0.98)	-				-			

*Adjusted for frequency of outpatient visits, age, sex, income, region, antidepressant, antipsychotic, anxiolytic, stabilizer, treatment type, CCI, year, medical institution type, and disease type

†Other psychotherapy includes continuous sleep therapy and psychiatric social work

The results of the logistic regression analysis analyzing the effect of COC on the likelihood of hospitalization by the type of medical institution visited for outpatient services and the type of disease diagnosed are depicted on Table 3. The main trends found were generally maintained. In tertiary hospitals, the medium (OR 0.22, 95% CI 0.13–0.37) and high COC groups (OR 0.10, 95% CI 0.06–0.17) had lower odds of hospitalization than the low COC group. Similar tendencies were found in secondary hospitals in which decreased odds of hospitalizations were present in the high (OR 0.61, 95% CI 0.39–0.96) compared to the medium COC group. In primary clinics, individuals with high COC (OR 0.19, 95% CI 0.06–0.62) again showed reduced likelihoods than those with low COC. In terms of disease type, the trends presented in Table 2 were again sustained, although statistical significance was only found in individuals with unipolar disorder (medium COC group: OR 0.33, 95% CI 0.21–0.52; high COC group: OR 0.14, 95% CI 0.09–0.21).

**Table 3:** Factors associated with hospital admissions by institution and disease type.

		OR*	95% CI		
**Type of medical institution**					
** Tertiary hospital**	Low	1.00			
	Medium	0.22	(0.13	-	0.37)
	High	0.10	(0.06	-	0.17)
** Secondary hospital**	Low	-			
	Medium	1.00			
	High	0.61	(0.39	-	0.96)
** Primary clinic**	Low	1.00			
	Medium	0.48	(0.15	-	1.61)
	High	0.19	(0.06	-	0.62)
**Type of disease**					
** Unipolar disorder**	Low	1.00			
	Medium	0.33	(0.21	-	0.52)
	High	0.14	(0.09	-	0.21)
** Bipolar disorder**	Low	-			
	Medium	1.00			
	High	0.87	(0.56	-	1.35)

*Adjusted for frequency of outpatient visits, age, sex, income, region, antidepressant, antipsychotic, anxiolytic, stabilizer, treatment type, CCI, year, and medical institution type/ disease type

## Discussion

The findings of this study reveal an association between COC and likelihoods of hospitalization in patients diagnosed with unipolar and bipolar disorder as individuals with higher COC showed reduced odds of hospitalization. As this study calculated COC for outpatient services during the first year of diagnosis and recorded whether hospitalizations took place in the year after COC measurement, hospitalizations may reflect patient outcomes. Specifically, the results infer the importance of COC in managing mood disorder patients as individuals in the medium and high COC groups exhibited gradationally lower odds of hospitalizations than individuals in the low COC group. The presented results are in line with previous findings which report that care coordination is associated with hospitalizations [17]. Beforehand, a study on outpatients with over two annual medical visits conveyed that patients with perfect continuity have lower risks of hospitalization within one year [18]. Another study focusing on elderly men discovered that groups with good continuity have lower admission rates [19]. As for studies conducted on East Asia, findings have identified that depressive symptoms may be a risk factor for increased hospitalization [20, 21]. This study confirms a relationship between COC and hospitalization in Korean unipolar and bipolar patients, suggesting the importance of providing an effective psychiatric patient management system to improve patient outcomes in the Asian population, including those in countries that lack a personalized general practitioner based primary care system.

The association between COC and hospitalization was generally maintained regardless of diagnosis and the type of medical institution visited for outpatient services. Trends show that the degree of difference was strongest in the tertiary hospital group, followed by the primary clinic group and the secondary hospital group. This tendency may have resulted as people experiencing poor continuity in primary care are more likely to contact higher level medical institutions [22]. However, it must also be taken into account that Korea lacks a general practitioner system, with patients being able to freely receive care from higher level institutions without a referral. Hence, the found relationship between COC and hospitalization suggests a possible need to monitor the health care utilization patterns of mental illness patients at all levels of medical institution.

With regard to all-cause mortality, previous findings have demonstrated the protective effects of improving longitudinal COC in reducing all-cause mortality of bipolar disorder and major depressive disorder patients [8, 23]. However, the association between COC and all-cause mortality did not show statistical significance in this study. The lack of statistical significance may have resulted as whereas this study only included participants diagnosed with unipolar or bipolar disorder, most other studies targeted mental disorder patients in general, including schizophrenia patients known to show exceptionally higher mortality rates.

The results of this study favor an association between COC and suicide mortality as individuals with better COC exhibited reduced risks of suicide mortality. Previous studies conducted in the United Kingdom (UK) and the US reported that better continuity may be associated with lower risks of suicide death, which is important as bipolar and major depressive disorder patients are known to exhibit the highest risk of suicide [24, 25]. As Korea ranks first among the Organization for Economic Cooperation and Development (OECD) countries in suicide rate, with suicide being the fifth leading cause of death nationally, this study offers insights by suggesting a possible association between improved COC and reduced suicide risk [26, 27]. The findings are also noteworthy as suicide mortality has been consistently rated high for patients with psychoses [28].

This study is not without its limitations. First, unipolar and bipolar mood disorders were classified only based on the ICD-10 codes. Other standard classification systems, including the Diagnostic and Statistical Manual of Mental Disorders (DSM) criteria, could not be utilized due to data limitation. Hence, inaccuracies may have resulted from different individuals being involved in the process of diagnosis recording. Second, date of death was only provided up to year and month by the KNHIS to protect personal information. Third, the number of included covariates were limited as the primary purpose of collecting and utilizing the NHI data is for reimbursement. Hence, the possibility of unmeasured confounding cannot be ruled out. Fourth, this study only calculated provider COC based on the UPC index. Thus, aspects such as the quality of provider-patient relationship or coordination of care were not incorporated. Last, this study could not adjust for mental illness severity due to data limitation. However, this study did take into consideration mental illness diagnosis, volume of annual outpatient visits, physical comorbidities, pharmaceuticals, and psychotherapy as covariates to partially cope for this limitation. Furthermore, only newly diagnosed individuals were included in the study population. Future studies improving the limitations stated above are needed to provide further insights.

## Conclusions

The findings of this study indicate that COC after first diagnosis of unipolar or bipolar depressive disorder is associated with subsequent hospitalization and suicide mortality. The results reveal the potential benefits of maintaining better psychiatric care treatment continuity in improving outcomes of mental disorder patients. Taking into account the fact that major depressive disorder has been assessed by the World Health Organization (WHO) as one of the most burdensome diseases to society, efforts should be made to address psychiatric treatment continuity in the coming decades.
